# Radionuclide imaging and therapy directed towards the tumor microenvironment: a multi-cancer approach for personalized medicine

**DOI:** 10.1007/s00259-022-05870-1

**Published:** 2022-07-05

**Authors:** Circe D. van der Heide, Simone U. Dalm

**Affiliations:** Department of Radiology & Nuclear Medicine, Erasmus MC, Rotterdam, The Netherlands

**Keywords:** Tumor microenvironment, Radionuclide imaging and therapy, Cancer stroma, Pan-cancer therapy, Theranostics

## Abstract

Targeted radionuclide theranostics is becoming more and more prominent in clinical oncology. Currently, most nuclear medicine compounds researched for cancer theranostics are directed towards targets expressed in only a small subset of cancer types, limiting clinical applicability. The identification of cancer-specific targets that are (more) universally expressed will allow more cancer patients to benefit from these personalized nuclear medicine–based interventions. A tumor is not merely a collection of cancer cells, it also comprises supporting stromal cells embedded in an altered extracellular matrix (ECM), together forming the tumor microenvironment (TME). Since the TME is less genetically unstable than cancer cells, and TME phenotypes can be shared between cancer types, it offers targets that are more universally expressed. The TME is characterized by the presence of altered processes such as hypoxia, acidity, and increased metabolism. Next to the ECM, the TME consists of cancer-associated fibroblasts (CAFs), macrophages, endothelial cells forming the neo-vasculature, immune cells, and cancer-associated adipocytes (CAAs). Radioligands directed at the altered processes, the ECM, and the cellular components of the TME have been developed and evaluated in preclinical and clinical studies for targeted radionuclide imaging and/or therapy. In this review, we provide an overview of the TME targets and their corresponding radioligands. In addition, we discuss what developments are needed to further explore the TME as a target for radionuclide theranostics, with the hopes of stimulating the development of novel TME radioligands with multi-cancer, or in some cases even pan-cancer, application.

## Introduction

In the last decades, it has become clear that solid tumors are more than a collection of malignant cells. The extracellular matrix (ECM) and host-derived, but altered, stromal cells are an important part of the tumor, and together form the tumor microenvironment (TME). Although the exact composition of the TME varies between patients, there is a general overlap in TME phenotypes among individuals [1, 2]. The TME can encompass more than half of the total tumor mass; it consists of different cellular components, i.e., tumor-infiltrating lymphocytes (TILs), tumor-associated macrophages (TAMs), cancer-associated fibroblasts (CAFs), cancer-associated adipocytes (CAAs), and endothelial cells of the neo-vasculature, that are embedded in the ECM. In addition, the TME is characterized by the occurrence of altered processes, such as acidity, altered metabolism, and hypoxia [3, 4]. Altogether, the TME is a mixture of anti- and pro-tumorigenic factors, and a shift in the balance towards a pro-tumorigenic TME will promote cancer growth and metastasis formation. In addition, the TME can form a protective barrier around cancer cells, hampering optimal drug delivery, hereby playing a crucial role in therapy resistance [5].

In nuclear medicine, radioligands can be used for targeted radionuclide imaging and targeted radionuclide therapy (TRT). These radioligands exploit biomarkers that are overexpressed on cancer cells, but not or only in low levels on healthy tissues, for the precise delivery of radioactivity. Nuclear imaging is relevant for disease staging, therapy selection, and treatment monitoring. Single-photon emission computed tomography (SPECT), using ligands radiolabeled with γ-emitting radionuclides (e.g., ^99m^TC, ^111^In, ^123/125^I), or positron emission tomography (PET), accomplished with positron emitters (e.g., ^18^F, ^68^Ga and ^60/64^Cu) is used, often in combination with computed tomography (CT) or magnetic resonance imaging for anatomical reference. TRT allows for internal irradiation of cancer cells. For this, ligands coupled to cytotoxic alpha- or beta-emitting radionuclides (e.g., ^225^Ac or ^177^Lu and ^90^Y respectively) are used. These therapeutic radionuclides can induce DNA damage, ultimately leading to cell death. Ideally, the same ligand, only differing in radioisotope, can be used for both imaging and therapy (i.e., theranostics).

The most successful clinically applied radioligand is [DOTA-Tyr^3^]octreotate (DOTATATE). DOTATATE, labeled with lutetium-177 or gallium-68, is EMA- and FDA-approved for theranostic application in somatostatin receptor type-2 expressing neuroendocrine tumors [6]. Thus, its use is limited to a specific subset of patients with overexpression of this receptor, as is the case for the majority of radioligands. More patients could benefit from theranostic radioligands when tumor-specific, yet more universally expressed biomarkers are discovered. Biomarkers present in the TME are very interesting for this purpose. The dynamic interactions between cancer cells and the surrounding stroma can make it challenging to find a suitable TME target. Nevertheless, when compared to healthy tissue stroma, these dynamics lead to the occurrence of TME-specific processes and the expression of TME-specific biomarkers, which can be exploited for precise tumor targeting [7]. Furthermore, genetic studies have shown that even though TME cells differ in gene expression from their healthy counterparts, the mutation rate in TME cells is low, and therefore these cells are, in general, less genetically unstable than cancer cells. For this reason, TME cells often provide more stably expressed biomarkers [8].

TME-directed radioligands have been developed and are evaluated in preclinical and clinical studies. Regarding nuclear imaging, this can be of value for tumor characterization, disease staging, patient selection (for non-nuclear medicine–based therapies, as well as for TRT), and treatment monitoring. Multiple non-radioactive TME-targeted anti-cancer therapies are in clinical trials or approved, and here nuclear imaging, often using a radiolabeled variant of the treatment compound, can offer a non-invasive method for patient selection. In addition, TRT can offer a personalized and precise treatment option for more patients.

**Fig. 1:**
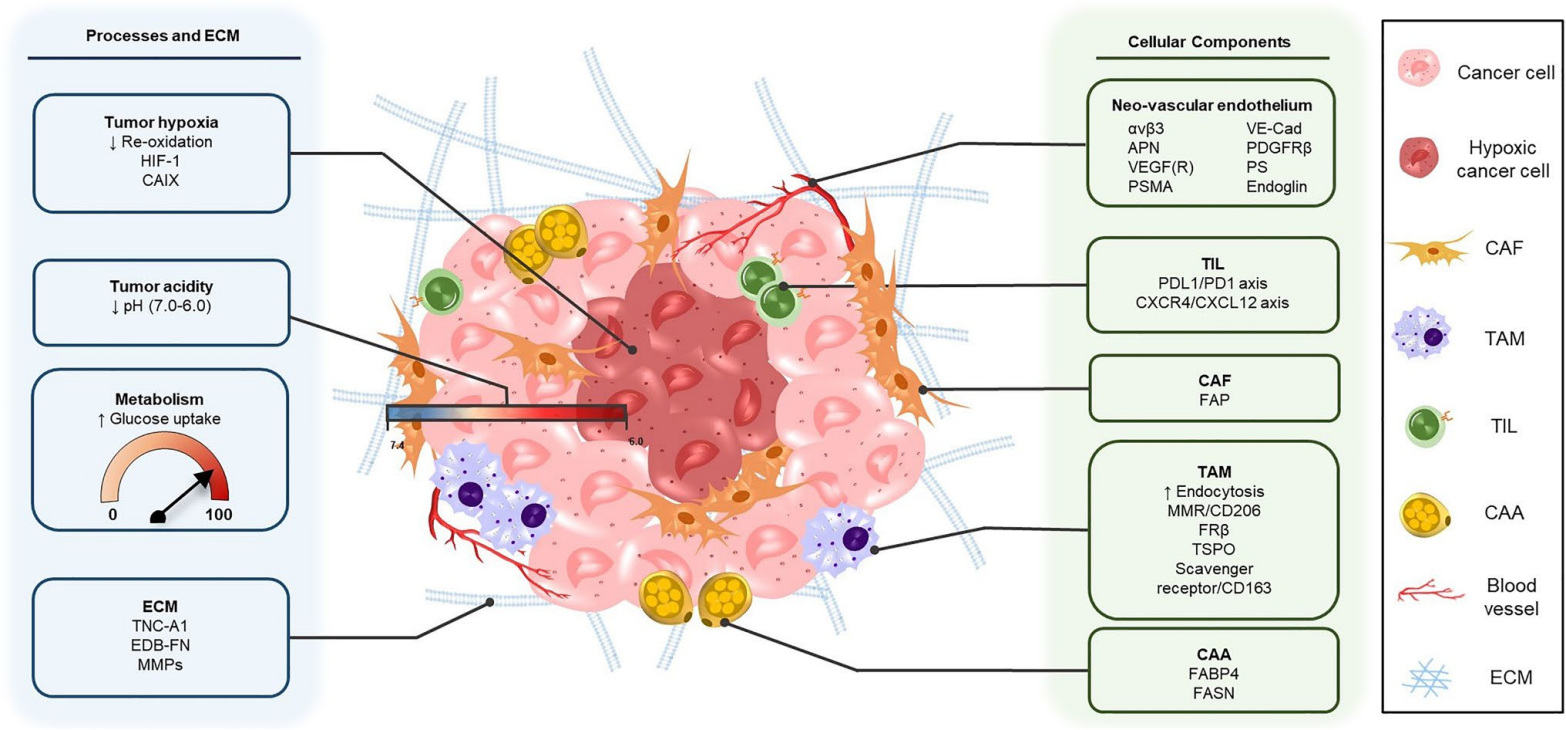
Targets in the TME suitable for radionuclide imaging and/or therapy. This includes altered TME processes and the EMC (left) and cellular components of the TME (right)

In this review, we will give an overview of the TME targets in solid tumors and their most relevant corresponding radioligands for imaging and therapy. We distinguish between targeting the altered TME processes and ECM, including molecular markers expressed as a consequence of these processes, and targeting the cellular components of the TME (Fig. 1). Hereby, we will provide insights into the current status of radionuclide imaging and therapy directed at the TME. Furthermore, we will discuss the challenges that need to be overcome to accelerate this specific field of nuclear medicine, with the goal of promoting the development and application of novel TME-targeting radioligands, with potential multi-cancer, or even pan-cancer, application.

## Targeting altered TME processes and the ECM

### Hypoxia

Hypoxia, a status of low oxygen levels, is a common feature of the TME [9]. It induces genetic instability and affects gene expression, leading to an aggressive phenotype and treatment resistance [10, 11]. Despite its clinical relevance, it is not routinely measured in the clinic due to the lack of a non-invasive method. Therefore, PET and SPECT imaging radioligands directed at the low oxygen levels in the TME, carbon anhydrase IX (CAIX), and hypoxia-induced factor-1 (HIF-1) have been developed and evaluated. The latter two are molecular markers that are expressed on TME cells as a result of the hypoxic TME status.

Low oxygen levels have mainly been targeted with nitroimidazole-based radioligands. In normoxic conditions, nitroimidazoles are reduced after which they are immediately re-oxidized. In contrast, in hypoxic conditions, these reduced nitroimidazoles cannot be re-oxidized, and as a consequence, they bind to intracellular macromolecules (e.g., DNA, proteins) in the hypoxic area. Based on this, nitroimidazole radioligands have been developed for SPECT or PET imaging, which show selective accumulation in hypoxic cells [12]. [^18^F]fluoromisonidazole (FMISO) is a first-generation radiolabeled nitroimidazole, of which increased uptake is correlated to a poor prognosis in multiple cancers [13]. Next-generation nitroimidazole agents include [^18^F]fluoroerythronitroimidazole (FETNIM), [^18^F]fluoroazomycin arabinoside (FAZA), [^18^F]-2-(2-itroimidazol-1-yl)-N-(3,3,3-mono/tri/pentafluoropropyl)-acetamide (EF1/3/5), [^18^F]-3-fluoro-2-(4-((2-nitro-1H-imidazol-1-yl)methyl)-1H-1,2,3-triazol-1-yl)propan-1-ol ([^18^F]-HX4), and [^18^F]fluoroetanidazole (FETA) [14, 15]. These next-generation radioligands show promising characteristics, especially [^18^F]FAZA, one of the most researched compounds. In preclinical animal studies, [^18^F]FAZA demonstrated a superior tumor-to-background ratio compared to the first-generation [^18^F]FMISO [16, 17]. Uptake of one of the other next-generation radioligands [^18^F]FETNIM had a prognostic value in patients, as was reported for [^18^F]FMISO [18]. A drawback is that most of the aforementioned next-generation nitroimidazoles show increased uptake in the bladder compared to first-generation [^18^F]FMISO, except for [^18^F]EF5 [19]. Furthermore, SPECT radioligands have been researched, but these were less successful than the above-mentioned radioligands for PET imaging [12].

Another class of hypoxia-targeted compounds that exploits a similar mechanism is the copper-labeled diacetyl-bis(N^4^-methylthiosemicarbazone) (Cu-ATSM). Cu-ATSM radioligands only get reduced in hypoxic conditions and as a consequence, like nitroimidazoles, are trapped in hypoxic cells [20]. [^60^Cu]Cu-ATSM has been clinically evaluated in several pilot studies with promising results. In 19 non-small cell lung cancer patients, [^60^Cu]Cu-ATSM PET could predict the response to chemotherapy and radiation therapy [21]. In another clinical study with 19 rectal carcinoma patients, [^60^Cu]Cu-ATSM uptake was found to predict tumor response to neo-adjuvant chemotherapy, and overall patient survival [22]. Cu-ATSM radioligands demonstrate advantageous pharmacokinetics and a higher detection threshold compared to FMISO [23]. Furthermore, the Cu-ATSM radioligands have more user-friendly properties than nitroimidazoles, including the longer half-life of copper isotopes compared to ^18^F [10]. Due to the benefits of 64-copper (e.g., high LET, short-range auger electron), [^64^Cu]Cu-ATSM is under preclinical and clinical investigation for both PET imaging (NCT03951337) and TRT (NCT04875871) in cervical cancer, rectal cancer, and glioblastoma [24, 25].

Two molecular markers, CAIX and HIF-1, are expressed on hypoxic cells that can be exploited for nuclear medicine–based interventions. CAIX expression is regulated by the transcription factor HIF-1, which is overexpressed in hypoxic tissues (pO_2_ of < 40 mmHg) [26]. To target CAIX, anti-CAIX monoclonal antibody (mAb) radioligands have been developed with varying degrees of success. To date, the most successful anti-CAIX radioligand in clinical trials is the chimeric mAb cG250 (Girentuximab). cG250 has been labeled with ^111^In, ^124^I, and ^89^Z for SPECT and PET imaging, as well as with ^90^Y and ^177^Lu for TRT [27]. Clinical pilot studies in metastatic renal cell carcinoma patients with ^111^In- and ^124^I-labeled cG250 demonstrated successful lesion detection [28, 29]. In addition, [^111^In]In-cG250 tumor uptake was predictive for [^177^Lu]Lu-cG250 accumulation [30]. In phase I/II clinical trials in renal cell carcinoma patients, both [^177^Lu]Lu-cG250 and [^90^Y]Y-cG250 demonstrated stabilization of disease, although myelotoxicity is a limiting factor [30, 31]. Another anti-CAIX mAb called “A3” has been preclinically evaluated; however, it demonstrated low tumor uptake and retention [27]. Antibodies have longer circulation times and slower tumor uptake than smaller compounds. To shorten the time between administration and imaging, and limit background noise, an improved blood clearance is necessary. This can be achieved by using smaller radioligands. In line with this, CAIX-targeted antibody fragments (Fabs), peptides, and small-molecule inhibitors have been developed. For example, the small immunoprotein (SIP) format of the aforementioned mAb A3, named SIP(A3). Unfortunately, similar to the full-size antibody, the SIP also demonstrated a low tumor uptake and retention [27]. In contrast, radiolabeled DTPA-cG250(FAb’)_2_ showed more promising results in preclinical studies. This Fab allowed for precise quantitative assessment of CAIX-positive tissue in mice xenografted with head and neck cancer cells [32]. The synthetic radiopeptides that have been produced were unfortunately not successful so far; they showed low tumor uptake and low CAIX specificity [33]. Concerning radiolabeled small-molecule inhibitors, a series of ^18^F- and ^99m^Tc-labeled benzene sulfonamide–based CAIX inhibitors have been tested in vitro by Lu et al. [34] for respective PET and SPECT imaging. These radiolabeled inhibitors demonstrate high target affinity; however, in vivo studies are needed to determine tumor uptake and retention, and tumor-to-background ratios. Another radiolabeled benzene sulfonamide CAIX inhibitor is ATS-DTPA-In[In^111^], which showed promising tumor-to-background ratios in a colorectal cancer xenograft mouse model [35]. In addition, Lau et al. [36] created three sulfonamide-based inhibitors with potent CAIX inhibition, and when radiolabeled demonstrated good imaging potential in tumor xenografted mice. More recently, affibodies targeting CAIX have been developed. The radiolabeled affibody ZCAIX:1, and its variations ZCAIX:2 and ZCAIX:3, showed promising results in preclinical models [37, 38]. [^99m^Tc]Tc(CO)_3_-(HE)_3_-ZCAIX:2 showed to be the most promising because of its high tumor uptake [39]. The ^111^In-labeled variant of this affibody, [^111^In]In-DOTA-ZCAIX:2, showed a twofold higher tumor uptake in renal cell carcinoma xenografted mice, outperforming the uptake of the aforementioned [^111^In]In-DTPA-G250(Fab’)_2_ [37]. However, the same group found that despite the high tumor uptake of [^111^In]In-DOTA-ZCAIX:2, the mAb [^111^In]In-DTPA-G250 still showed the best tumor-to-background ratio in this preclinical setting [40].

Next to targeting CAIX, the upstream transcription factor HIF-1 can be targeted. To target HIF-1-positive tumor cells, a ^123/125^I-labeled fusion protein, PTD-ODD-SAV (IPOS), has been developed. IPOS has the same oxygen-dependent degradation domain as HIF-1 and therefore follows the same degradation pathway. Thus, radiolabeled IPOS is degraded in normal tissues, and accumulates in hypoxic cells. [^123^I]-IPOS showed high and selective tumor accumulation in hypoxic and HIF-1 active tumors in preclinical studies [41]. Another probe based on this degradation domain is [^125^I]I-DKOP30, which is a radiopeptide with improved immunogenicity and pharmacokinetics compared to [^125^I]-IPOS. Similar to IPOS, [^125^I]I-DKOP30 showed tumor accumulation coinciding with hypoxic regions in animal studies [42].

For clinical implications, it is important to note that necrosis is common in hypoxic areas. Because necrotic cells do not have radioligand uptake, they decrease the PET and SPECT signal of hypoxia-targeted radioligands, which affects image interpretation [43].

### Acidity

In many cancers, a low pH is observed in the interstitial space of the tumor (pH 6.3–7.0). This is linked to increased production of lactic acid, which is a consequence of increased glucose uptake in cancer cells, the so-called Warburg effect [44]. The lactic acid load is transported out of the cancer cells, however, cannot be transported out of the tumor due to poor blood flow, which results in an acidic TME [45]. The previously described state of hypoxia in the TME can trigger the Warburg effect, thus hypoxia is one of the causes of an acidic TME. Studies have shown that a low extracellular pH is associated with treatment resistance, a less favorable prognosis, and promotes metastasis formation [46, 47]. It is therefore clinically relevant to develop radioligands for imaging acidity in tumors.

The pH-low insertion peptides (pHLIPs) are promising pH-sensitive ligands. These pHLIPs change their shape under low pH conditions, which allows them to penetrate the cellular membrane and accumulate in tumor cells [48]. Multiple pH-sensitive pHLIP constructs such as WT, Var3 &Var7 have been developed and labeled with ^64^Cu and ^18^F. In preclinical evaluation, the NO2A-cysVar3 was proved to be the best construct, yielding a high tumor uptake at 4 h post injection (p.i.) when labeled with ^18^F and ^64^Cu [49]. The Var7 pHLIP was also labeled with iodine-125 by Yu et al. [50]. [^125^I]I-pHLIP showed rapid tumor uptake in mice bearing breast cancer xenografts, but unfortunately tumor uptake and retention were lower than that observed for ^18^F- or ^64^Cu-labeled Var-3. A modified ^18^F-labeled Var3 construct has been evaluated in a phase I clinical trial in breast cancer patients (NCT04054986), of which the results are not yet published. Even though the results of pHLIP imaging are promising, radiolabeling of these peptide inserts can be challenging due to their large size, and more experience is needed to accelerate radiolabeled pHLIP development [51].

**Fig. 2:**
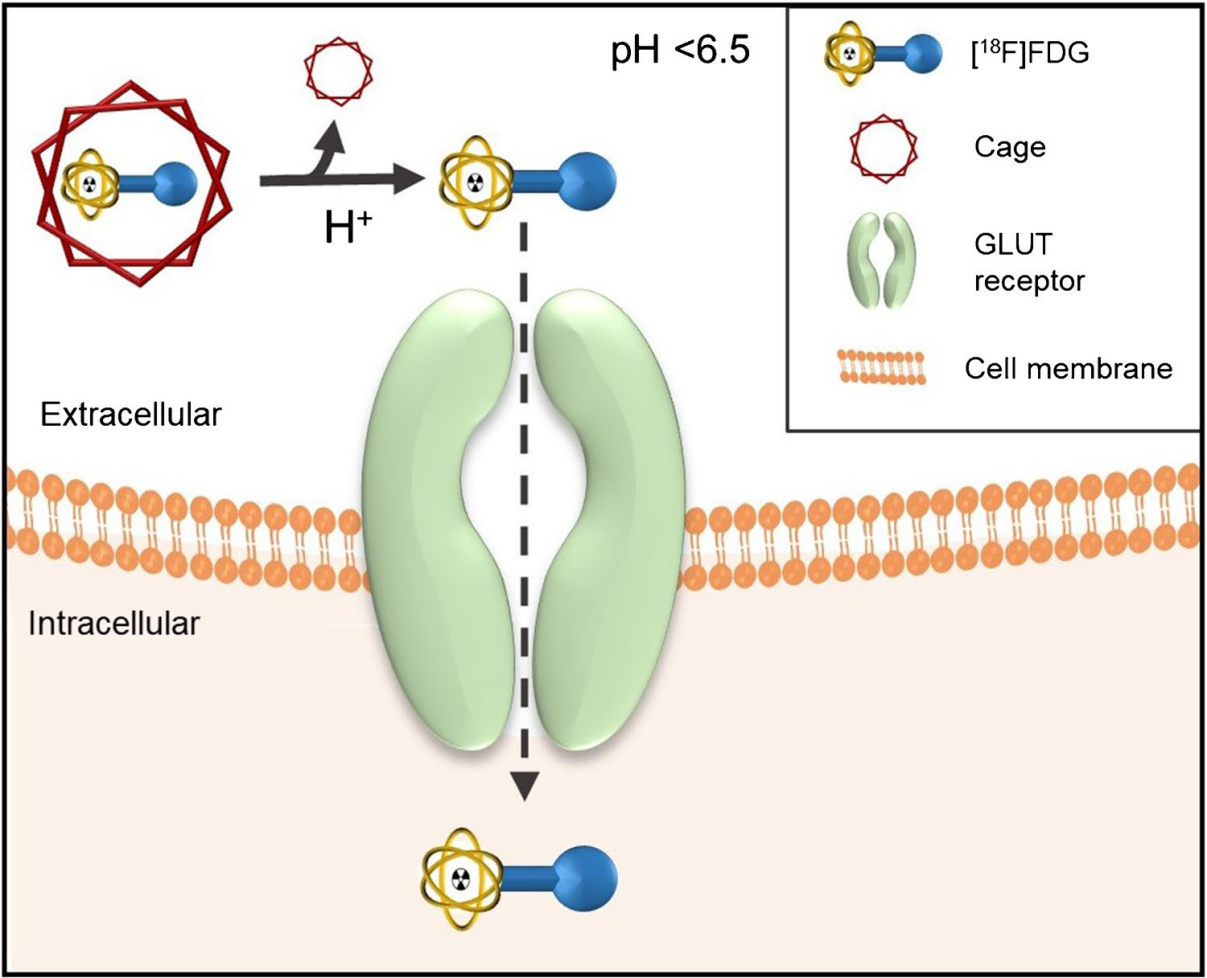
The concept of using [^18^F]FDG amine for pH-dependent (pH < 6.5) release of [^18^F]FDG. The [^18^F]FDG amine cage is cleaved at low extracellular pH. Consequently, [.^18^F]FDG is released, which then enters tumor cells through the GLUT receptor, enabling visualization of acidic lesions on PET scans. The figure is based on Flavell et al. [52]

Preclinical pH-responsive PET imaging has also been accomplished with [^18^F]-fluorodeoxyglucose ([^18^F]FDG) amines. The amine functions as a cage, which is cleaved at low pH to release [^18^F]FDG, which can enter the tumor cells to be imaged with PET (Fig. 2). In vivo stability is an issue with this compound, as an unstable cage can result in uptake in non-acidic tissues, herewith decreasing the accuracy of the method [52]. Nevertheless, this approach is interesting as it can potentially be applied for the pH-dependent release of other radioligands. Another pH-responsive example is the malonic acid derivatives, which become lipophilic and penetrate the cell membrane only at a pH < 6.5. The most promising malonic acid derivative is 2-(4-[^123^I]iodophenethyl)-malonic acid ([^123^I]-IPM), which showed reliable pH-dependent uptake in tumor cells in a preclinical setting [53].

### Metabolism

Tumors arise from the unlimited proliferation of cancer cells, which results in an increased need for ATP, amino acids, and other building blocks of the cell. To account for this, cancer cells exhibit an altered metabolism [54]. Differences in metabolic activity arise heterogeneously throughout the tumor, and this has been used as a target for nuclear imaging to monitor changes in the TME after treatment [55].

The gold standard for cancer imaging is the radiolabeled glucose analog [^18^F]FDG PET. It relies on the increased glucose uptake in cancers, due to the previously explained Warburg effect, and in line with this tumors have a high uptake of [^18^F]FDG [56]. Research has shown that the TME can affect [^18^F]FDG uptake, and thus tumor imaging. Especially, inflammation and hypoxia, which both result in a high glucose demand and increased [^18^F]FDG uptake, as well as necrosis, associated with a relative low [^18^F]FDG uptake, alter the [^18^F]FDG PET signal [57, 58]. In addition, specific cell types such as CAFs, TAMs, and TILs can increase the [^18^F]FDG SUVmax on PET scans, due to increased proliferation and herewith associated increased glucose demand [59–61]. In line with the above, a higher SUVmax was observed in tumors with a higher number of tumor-promoting stromal cells. Furthermore, a study by Sasada et al. [62] demonstrated that [^18^F]FDG SUVmax together with Ki67 labeling and tumor size could be used to determine TIL score, which in turn predicted response to neo-adjuvant chemotherapy in breast cancer patients. Thus, ECM components can distort tumor [^18^F]FDG PET signal. Correct identification of the source of the [^18^F]FDG PET signal, if possible, is challenging. Additional information, e.g., coming from scans with complementary radioligands or biomarker analyses (e.g., IHC for cell-specific molecular markers), is needed for exact identification of the cellular source responsible for the [^18^F]FDG PET signal.

### Extracellular matrix

Tumor ECM differs significantly from healthy ECM in its stiffness and composition, which is the result of ECM remodeling [63]. Remodeling of the ECM is under the regulation of tenascin-C (TNC) and matrix metalloproteinases (MMPs), and increased stiffness is promoted by fibronectin (FN). TNC, MMPs, and FN stimulate cancer cell invasion and serve as ECM targets for radionuclide interventions.

**Fig. 3:**
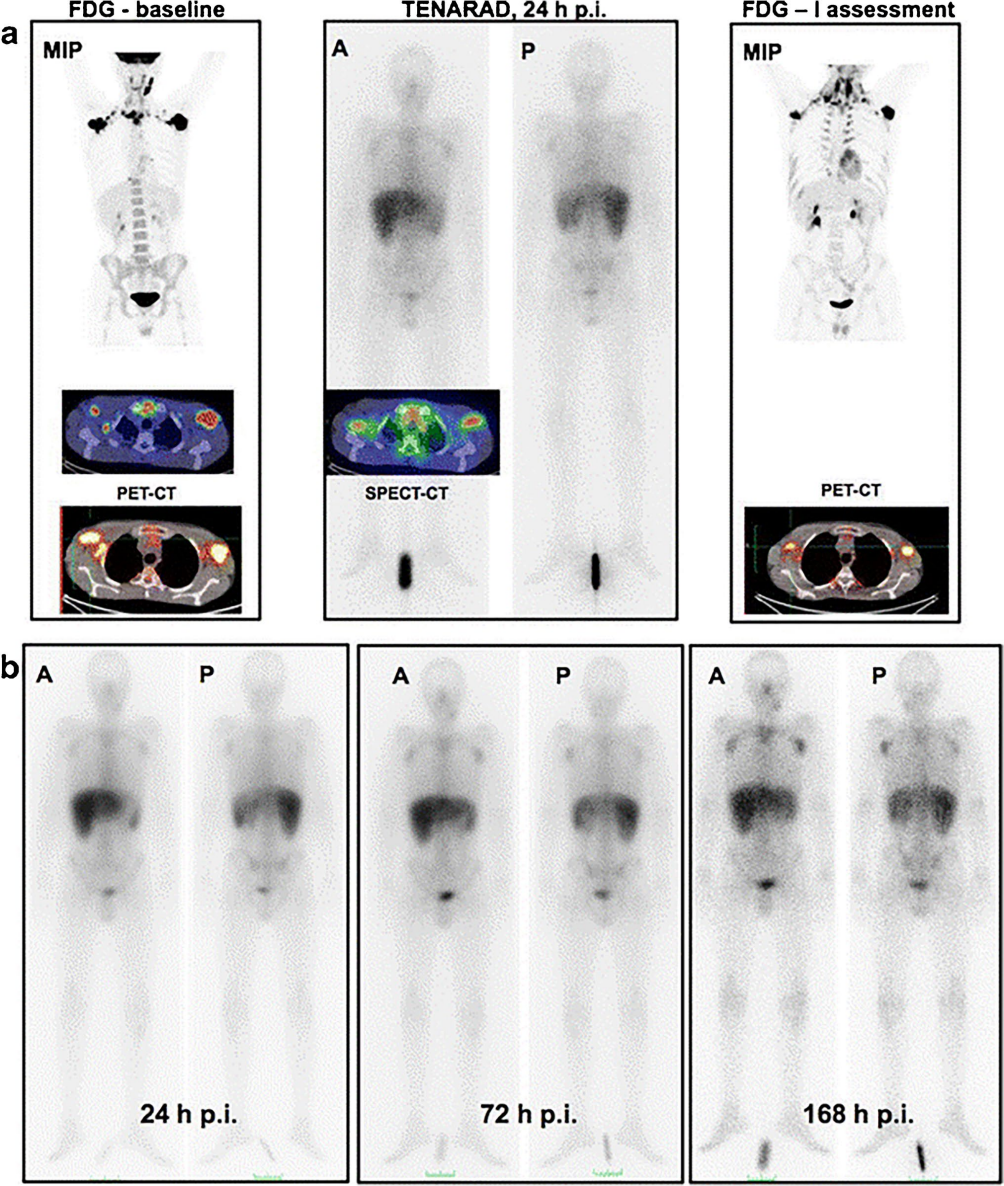
PET/CT scans of 1/5 responding Hodgkin’s lymphoma patients treated with the TNC-A1-targeting radioligand [^131^I]I-F16SIP (Tenarad). **a** Maximum intensity projection image of the baseline FDG PET/CT scan (left), the [^131^I]I-F16SIP SPECT/CT scan 24 h post-injection (middle), and the FDG PET/CT scan 4 weeks after [^131^I]I-F16SIP treatment (right). **b** Whole-body images at different time points after 3.33 GBq [^131^I]I-F16SIP administration, demonstrating uptake in tumor lesions. *A: anterior view*, *P: posterior view.* The figure was originally published in EJNNMI by Aloj et al. [65]. Radioimmunotherapy with Tenarad, a 131I-labeled antibody fragment targeting the extradomain A1 of tenascin-C, in patients with refractory Hodgkin's lymphoma (2014) Vol 41(5):867–877

TNC is an ECM component that is commonly expressed in healthy tissues. However, isoforms containing the A1 domain (TNC-A1) are involved in angiogenesis and are exclusively overexpressed in pathological conditions, including tissue remodeling, wound healing, and cancers [64]. TNC-A1 can be targeted with the specific mini antibody F16SIP (Tenarad). The iodine-124 and iodine-131 labeled variants of F16SIP can be used for TNC-A1-targeting radionuclide imaging and TRT. Using [^124^I]I-F16SIP PET, a tumor-specific signal was observed in 4 head and neck cancer patients. For TRT, [^131^I]I-F16SIP was evaluated in a phase I/II clinical trial with Hodgkin’s lymphoma patients. The radioligand was able to reduce lesion size and number of lesions in 5/8 Hodgkin’s lymphoma patients. Figure 3 shows a representative example of a patient responsive to [^131^I]I-F16SIP treatment. Unfortunately, none of the patients demonstrated a lasting response. Furthermore, minor hematological toxicity was observed in most patients, and one patient even presented with severe toxicity [65, 66]. This needs further evaluation before larger clinical studies with [^131^I]I-F16SIP TRT are performed.

FN is the most abundant ECM protein [67]. FNs are key factors in communication between the ECM and cancer cells, and are especially overexpressed at the invasive front [68]. In particular, the FN isoforms contain the extradomain-A (EDA-FN) or extradomain-B (EDB-FN), which are absent in healthy adult tissues [69]. In addition, EDA-FN and EDB-FN interact with integrins αvβ1 and α5β1, involved in angiogenesis [70, 71]. EDB-FN can be targeted with mAb L19, or Fabs L19SIP, L19(ScFv)_2_, and AP39 [68]. All these L19 Fabs showed promising results for imaging EDB-FN in patients with various cancers, of which L19SIP demonstrated the best tumor-to-background ratios [72–74]. In a clinical trial in patients with extracranial brain tumor lesions, radiolabeled L19SIP theranostics was evaluated. In this study, [^124^I]I-L19SIP PET was used for patient selection for consecutive [^131^I]I-L19SIP treatment. [^124^I]I-L19SIP PET successfully identified patients that responded to consequent [^131^I]I-L19SIP treatment, which reduced lesion size as observed on [^18^F]FDG PET [75]. Next to these Fabs, radiopeptides targeting FN have been developed. Arnoldini et al. [76] synthesized [^111^In]In-FnBPA5, a radiopeptide that targets relaxed FN, to determine the stiffness of the ECM. The radiopeptide showed reasonable tumor uptake and retention in preclinical in vivo studies (4.74 ± 0.77% injected dose/gram (ID/g) 1 h p.i. vs. 3.59 ± 0.53%ID/g 24 h p.i.). However, excessive kidney uptake is a major limiting factor for this peptide (140.58 ± 18.10%ID/g 1 h p.i.). Another radiopeptide, ZD2-(^68^Ga-NOTA), showed improved tumor-to-kidney ratios in mice xenografted with pancreatic tumors, however, with a very low tumor uptake [69]. More recently, Jailkhani et al. [77] generated NJB2, a single-domain antibody (sdAb) against EDB-FN. NJB2 has a high affinity for EDB-FN, and [^64^Cu]Cu-NJB2 could successfully image early pancreatic cancer lesions and breast cancer metastases in mice. This was more effective than lesion detection with [^18^F]FDG.

**Fig. 4:**
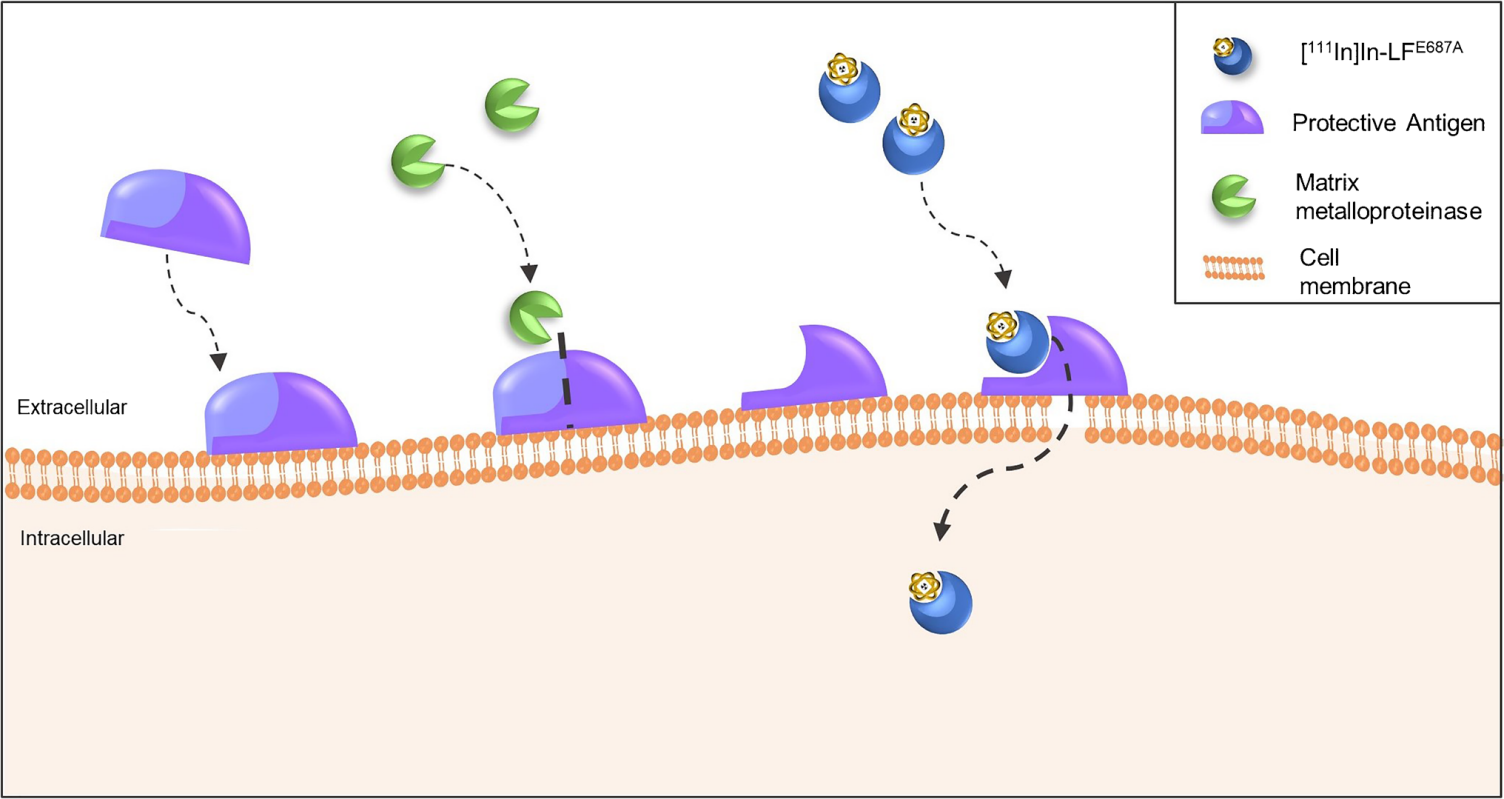
Pre-targeting system for imaging of ECM remodeling. Protective antigen binds to the cell membrane in the presence of MMP-2, -9, and/or -14 allowing to form a pore in the membrane. Hereafter, an indium-111 labeled LF.^E687A^ is administered which selectively binds to the cleaved protective antigen, and enters the cells via the created pore. The figure is based on Xavier et al. [84]

MMPs are also explored for radioligand targeting. MMPs play an important role in the degradation and remodeling of the ECM, and hereby they promote cancer progression [78]. For cancer treatment, various MMP inhibitors (MMPIs) have been developed, and targeted radionuclide imaging can help identify the expression of specific MMPs or MMP families in cancer for treatment selection. For this purpose, it is important to design radiolabeled MMPIs that are highly selective for a specific MMP or MMP family. A variety of radiolabeled MMPIs has been designed for imaging, including ^18^F-labeled inhibitors against MMP-2, -8, -9, or -13 [79], ^11^C-labeled analogs of the MMP-3 inhibitor CGS 27023A [80], a specific MMP-13 inhibitor labeled with ^11^C, ^18^F, and ^68^Ga [81], and a series of fluorinated MMP2/MMP9-family inhibitors [82]. These radioligands show promising affinities for their corresponding MMPs; however, further studies are needed to determine the in vivo biodistribution and tumor uptake. Little in vivo preclinical and clinical research has been performed with radiolabeled MMPs, because no radiolabeled MMP currently has an optimal balance between target affinity, hydrophilicity, and intrinsic albumin affinity, for optimal tumor accumulation and tumor-to-background ratios [83]. Another approach to image areas in the TME with high ECM remodeling is by using an MMP cleavable pre-targeting system. This is performed with a modified bacterial protein, called protective antigen, which attaches to the cancer cell membrane and creates a pore, only in the presence of MMP-2, -9, and/or- 14. The modified protective antigen is administered first. Hereafter, the ^111^In-labeled compound (i.e., LF^E687A^) is administered that selectively binds to the cleaved protective antigen, resulting in [^111^In]In-LF^E687A^ internalization through the created pore (Fig. 4). This pre-targeting system demonstrated MMP-dependent tumor accumulation and rapid blood clearance of [^111^In]In-LF^E687A^ in animal studies. However, due to hepatic clearance of the compound, there was high accumulation in the liver and spleen, resulting in a suboptimal tumor-to-background ratio [84].

### Summary: targeting TME processes and the ECM

A number of radioligands targeting altered TME processes, and the molecular markers that are expressed as a consequence hereof, and the ECM have been developed and evaluated (Table 1). As altered oxygen levels, acidity, and metabolism are a prerequisite for tumor growth, they occur in many cancer types. Therefore, these processes offer semi-universal targets for nuclear medicine–based interventions. Both TME hypoxia and acidity induce treatment resistance and have a negative prognostic value [11, 47]. Nuclear imaging can provide a non-invasive method for monitoring these processes for treatment selection. A number of the aforementioned radioligands (i.e., nitroimidazoles, pHLIPs, [^18^F]FDG) have high clinical potential to aid herein [47, 55]. For imaging of hypoxia and acidity, more research is needed to understand the influence of necrosis and tumor heterogeneity on hypoxia- and pH-targeted radioligand uptake [43]. This will help comprehend radioligand distribution and improve image interpretation. Regarding [^18^F]FDG PET for imaging TME components, more research is needed to determine whether and how to attribute the source of the [^18^F]FDG PET signal to cancer cells or TME cells. Since [^18^F]FDG is already approved, clinical implementation for novel applications is relatively straightforward.

**Table 1:** An overview of the targets expressed as a result of altered TME processes and the tumor ECM, with a number of their corresponding radioligands

Source	Target	Compound	Research phase	Reference
**Hypoxia**	Nitroimidazole analogs	FMISO FAZA FETNIM Cu-ATSM FETA EF1/3/5 FRP170 HX4 N4-NIM	Approved Approved Clinical trial Clinical trial Preclinical Preclinical Preclinical Preclinical Preclinical	[12, 14, 15, 24]
	CAIX	cG250 cG250-F(Ab’) A3/SIP(A3) CAIX inhibitors ZCAIX:1/2/3/4	Clinical trial Preclinical Preclinical Preclinical Preclinical	[30, 31, 34, 36, 37]
	HIF-1	IPOS (m)DKOP	Preclinical Preclinical	[41, 42]
**TME acidity**	Low extracellular pH (6.0–7.0)	pHLIPs FDG amine Malonic acid derivatives	Clinical trial Preclinical Preclinical	[52, 53]
**Metabolism**	Elevated glucose uptake	FDG	Approved^a^	[59, 60, 62]
**Extracellular matrix**	TNC-A1	F16SIP (Tenarad)	Clinical trial	[64, 65]
	EDB-FN	L19 L19SIP L19(ScFv)_2_ AP39 FnBPA5 ZD2-NOTA NJB2	Clinical trial Clinical trial Clinical trial Preclinical Preclinical Preclinical Preclinical	[69, 73, 76, 77]
	MMPs	MMPIs Protective antigen + LF^E687a^	Preclinical Preclinical	[78, 84]

^a^In clinical use for tumor imaging, not for specific TME imaging

Radioligands targeting hypoxia and acidity have potential multi-cancer imaging applications, since the majority of cancers have an acidic and hypoxic TME. Targeting of these altered processes for TRT is relatively unexplored, indicating that they might be not suitable for this purpose. In contrast, the specific molecular biomarkers HIF-1, CAIX, and ECM targets TNC and EDB-FN have been more explored for TRT. However, only a few preclinical and clinical studies are available on HIF-1 and ECM-targeting radioligands, possibly due to the lack of target specificity and poor pharmacokinetics of most of these radioligands.

Concerning the molecular markers, HIF-1 and CAIX, radioligands targeting the latter have been more clinically evaluated; however, CAIX is not always expressed in hypoxic cells, making it less suitable for a multi-cancer approach [85]. In line with this, it is important to identify which cancer (sub)types express CAIX, to select patient subsets that can be eligible for CAIX-targeted nuclear interventions. In addition, different isoforms of both HIF-1 and CAIX can exist, and isoform-specific compounds can be valuable [85]. A nuclear medicine–based theranostic strategy can be promising to select patients with CAIX expression that will benefit from CAIX TRT. Other compounds that are especially relevant for theranostics are the radiolabeled MMPIs. These can play an important role in patient selection for therapy with the corresponding unlabeled MMPI. The development of specific MMP-targeting radioligands for which radioligands are not available yet is needed for improved patient selection and MMPI treatment. Furthermore, innovative strategies, such as the MMP-dependent radioligand uptake and the caged-[^18^F]FDG, are important to stimulate the development of novel TME-targeting radioligands for nuclear imaging and TRT.

In conclusion, TME-specific processes and the ECM provide semi-universal cancer targets for nuclear imaging. These processes and their corresponding molecular markers are not extensively explored for TRT. Targets that are more promising for both targeted radionuclide imaging and TRT can be found on the cellular TME components that will be discussed in the next section.

## Cellular components of the TME

### Cancer-associated fibroblasts

CAFs are the most abundant cell type in the TME [86]. They are heterogeneous and originate from different cellular sources (e.g., fibroblasts, endothelium, epithelium); together with the disease stage, this determines if they are tumor-promoting or tumor-restraining [87]. CAFs are characterized by the expression of fibroblast activation protein-α (FAP), a membrane-bound protease that is expressed in over 90% of all epithelial carcinomas [88]. In addition, FAP is almost exclusively expressed in chronic inflammation, wound healing, and cancer, thus absent from healthy tissues. FAP-expressing CAFs can promote tumor growth and invasion, and are often associated with poor prognosis [89]. Apart from CAFs, FAP expression has been found on colon, breast, bone, lung, ovarian, and pancreas cancer cells [90, 91]. Together, the abundance and the unique expression of FAP make FAP-expressing CAFs an appealing target for nuclear imaging and TRT with potential pan-cancer application.

**Fig. 5:**
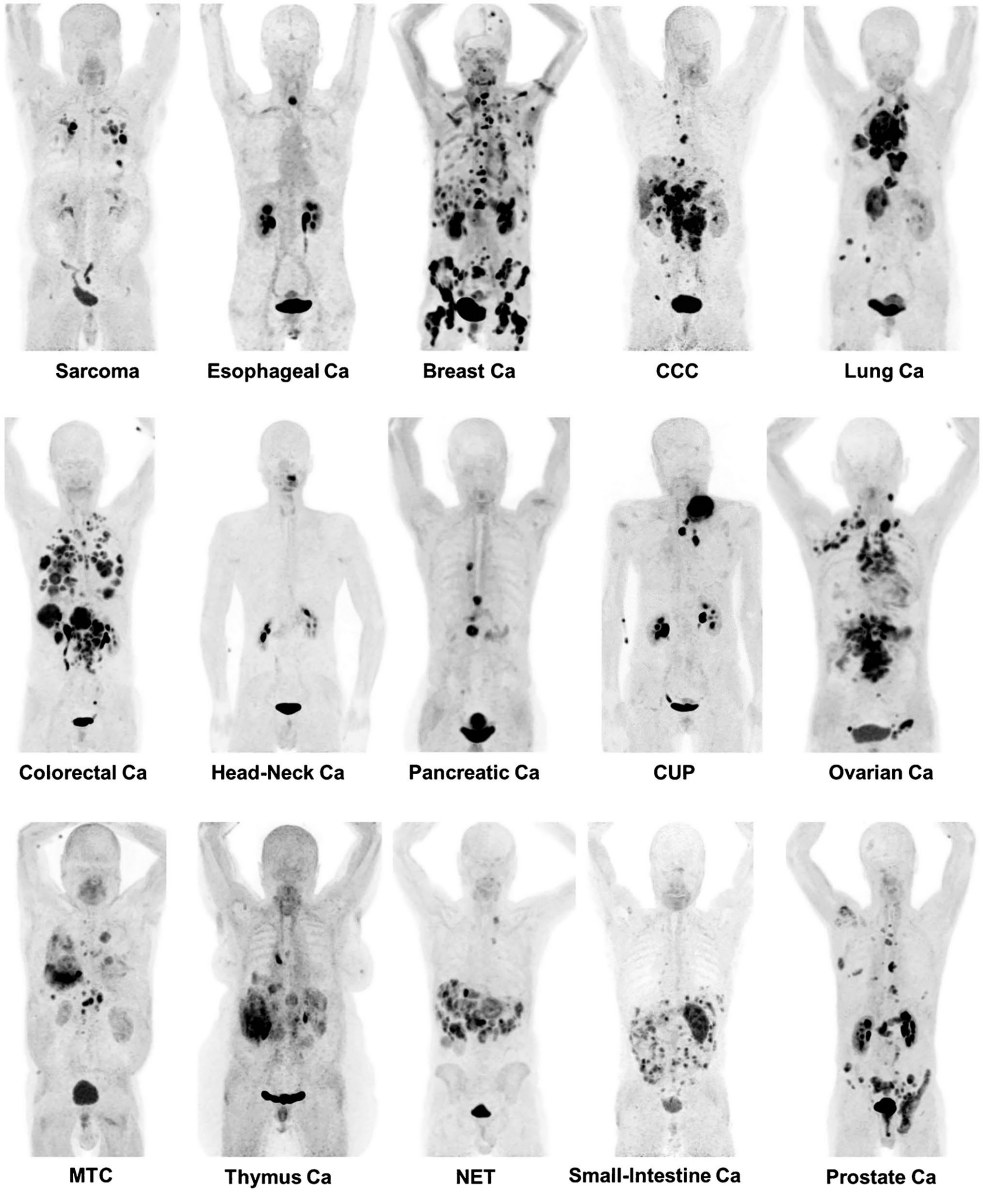
[^68^Ga]Ga-FAPI-04 PET/CT scans in 15 different cancer types demonstrating the pan-cancer potential of FAP-targeting radionuclide imaging. *Ca* cancer; *CCC* clear cell carcinoma; *CUP* carcinoma of unknown primary; *MTC* medullary thyroid cancer; *NET* neuroendocrine tumor *NSCLC* non-small cell lung cancer. The figure was originally published in JNM. Kratochwil et al. [95]. ^68^Ga-FAPI PET/CT: Tracer Uptake in 28 Different Kinds of Cancer (2019) Vol. 60(6): 801–805

FAP-targeting radioligands are an exciting recent development in the field of nuclear medicine. In recent years, these radioligands have shown promising initial results and have gained a lot of attention. Researchers of the University of Heidelberg were the first to report on FAP inhibitors (FAPIs) for radionuclide theranostics. A panel of radiolabeled FAPIs were developed by this group, of which FAPI-04, FAPI-46, and FAPI-74 were proved to be the most successful in clinical imaging studies [92–94]. PET/CT scans with FAPI-04 allowed for the diagnosis of 28 different tumor types in 80 patients (Fig. 5) [95], illustrating the potential pan-cancer application of the radioligand. In addition, FAPI-04 imaging outperformed [^18^F]FDG PET/CT in several cancer types; improved tumor-to-background ratios were observed with [^68^Ga]Ga-FAPI-04 [96, 97]. Although ideal for imaging, these radioligands unfortunately demonstrate short tumor retention, which makes them inadequate for treatment. FAPI-46 and FAPI-74 show improved tumor retention compared to the rest of the FAPI-series; however, the tumor retention remains suboptimal for treatment [98–100]. In pancreatic and glioma mouse models, efficacy studies with [^177^Lu]Lu-FAPI-46 and [^225^Ac]FAPI-46 accomplished tumor growth inhibition [101, 102]. However, in advanced-stage metastatic cancer patients, [^177^Lu]Lu-FAPI-04 demonstrated no treatment effect [103].

The compound with the highest reported FAP affinity so far is OncoFAP, an organic small natural FAP ligand, developed by Backhaus et al. [104]. The IC_50_ of ^nat^Ga-OncoFAP is 0.51 ± 0.11 nM, compared to an IC_50_ of 8.37 ± 0.71 nM for ^nat^Ga-FAPI-46. However, it has not been established whether the improved affinity results in increased tumor uptake and retention in vivo. Another novel compound, which was first reported by Baum et al. [105] and is currently under patent, is FAP-2286. The first results of [^177^Lu]Lu-FAP-2286 treatment in a group of metastatic cancer patients demonstrated high tumor uptake and reasonable retention, with stable disease in 2/11 patients after one treatment cycle [105]. FAP-2286 is currently in two ongoing clinical trials ([^68^Ga]Ga-FAP-2286 (NCT04621435) and [^177^Lu]Lu-FAP-2286 (NCT04939610) for evaluation of its imaging and therapy potential respectively, in various solid tumors.

In an attempt to improve the retention time of FAP-targeting radioligands, dimers have been developed. This includes dimers of the previously discussed FAPI-46 and OncoFap. The FAPI-46-based dimer, DOTA-2P(FAPI)_2_ was radiolabeled with gallium-68 and demonstrated significantly prolonged tumor retention compared to its monomer in a patient-derived xenograft mouse model (7.61 ± 0.64 vs. 3.81 ± 0.18%ID/g respectively). Moreover, a pilot study in three patients demonstrated a significantly higher SUVmax with the dimer compared to the monomer (SUVmax 1 h p.i.: 8.1–39.0 vs. 1.7–24.0, respectively). The increased SUVmax is most likely the result of longer blood retention of [^68^Ga]Ga-DOTA-2P(FAPI)_2_, compared to the monomer, which next to the tumor unfortunately also leads to a higher effective dose to healthy organs [106]. In addition, dimerization of OncoFap resulted in BiOncoFap. In a preclinical investigation, [^177^Lu]Lu-BiOncoFap demonstrated a longer retention time compared to [^177^Lu]Lu-OncoFap, and promising tumor-to-background ratios were observed for the dimer [107]. Two other FAP-dimers were developed based on the monomer SA.FAPI, the homodimers DOTA.(SA.FAPI)_2_, and DOTAGA.(SA.FAPi)_2_. [^68^Ga]Ga-DOTAGA.(SA.FAPi)_2_ had the best tumor uptake out of the two. Similar to what was reported for the other dimers, a clinical study with [^68^Ga]Ga-DOTAGA.(SA.FAPi)_2_ demonstrated higher tumor retention, as well as an increased blood pool accumulation compared to the monomer in six cancer patients [108].

### Tumor-associated macrophages

TAMs are the most prominent immune cell in the TME. They can exhibit a tumor-suppressing (M1) or tumor-promoting (M2) phenotype [109]. An abundance of TAMs in the TME is related to poor prognosis and disease progression in most cancers [110]. Even though research on TAMs has increased in the last decade, radioligands targeting TAMs are only slowly being developed. Possibly, due to the difficulty to identify markers specifically expressed by tumor-promoting M2 macrophages [111]. Targets that have been considered so far are the macrophage mannose receptor (MMR/CD206), the translocator protein (TSPO), the folate receptor-β (FRβ), the scavenger receptor (CD163), and the increased endocytic activity of TAMs.

MMR is overexpressed on M2 macrophages, making it a potent TAM target. However, its expression is not exclusive to TAMs. Other dendritic cells and phagocytic cells also demonstrate expression of MMR at lower levels [112]. The MMR-targeting compound [^99m^Tc]Tc-Tilmanocept (Lymphoseek®) has been FDA-approved for the identification of sentinel lymph nodes, and is under clinical investigation for imaging of TAMs in metastatic melanoma (NCT04663126) [112]. In the search for other MMR-targeting radioligands, sdAbs have successfully been radiolabeled and evaluated preclinically [109]. These sdAbs demonstrate high potential for nuclear imaging in mice, when labeled with ^99m^Tc, ^18^F, or ^68^Ga. Tumor uptake of radiolabeled NOTA-anti-MMR-sdAb was similar for all three aforementioned isotopes [113, 114]. Unfortunately, high uptake in the kidneys and MMR-expressing tissues (e.g., liver and spleen) was also observed. Of note, kidney uptake was drastically lower for the ^18^F-labeled anti-MMR-sdAb compared to the ^99m^Tc- and ^68^Ga-labeled compounds [109, 113, 114]. A different sdAb, [^68^Ga]Ga-NOTA-anti-MMR-VHH2, is currently under evaluation for PET imaging of TAMs in a phase I/II clinical trial in various solid tumors (NCT04168528). MMR targeting has also been studied using mannose-decorated liposomes (Man-Lips) carrying ^64^Cu. The Man-Lips demonstrated specific accumulation and high uptake in TAMs in a lung cancer mouse model. However, due to long retention in the blood, a long time interval between injection and imaging is needed to accomplish optimal tumor-to-background ratios [115]. [^18^F]FDM, which is an MMR-targeting radioligand based on [^18^F]FDG, has been created by replacing the D-glucose with D-mannose. [^18^F]FDM demonstrated 35% higher specific TAM uptake compared to [^18^F]FDG [116]. However, [^18^F]FDM showed equal uptake in inflamed tissues as in tumors [117].

TSPO is another biomarker explored for M2 radioligand targeting. TSPO-targeting radioligands have mainly been tested in glioblastoma, due to the overexpression of TSPO in activated microglial cells next to its expression in TAMs. In addition, TSPO expression is not limited to M2 macrophages and can also be found on other phagocytotic cells [118]. The first-generation TSPO-targeting radioligand [^11^C]-(R)PK11195 has been extensively researched for inflammation and tumor imaging, despite its low tumor-to-background ratio and lack of specificity [119]. To overcome these limitations, novel TSPO-targeting radioligands are being developed such as the acetamides PBR01, PBR06, and PBR28, of which the latter is the most promising [120, 121]. [^11^C]-PBR28 allowed for specific tracking of TAMs in a pancreatic cancer mouse model [122]. Moreover, [^11^C]-PBR28 has been clinically evaluated for detecting macrophage infiltration after treatment in glioblastoma and melanoma patients (NCT02431572). To our knowledge, the results of this study have not been published yet. Carbon-11 is impractical due to its short half-life (*t*_1/2_ = 20.38 min), and therefore an ^18^F-labeled PBR28 has been developed [120]. In line with this, other TSPO-targeting radioligands, DPA-714 and DPA-713, have been designed, which also allowed for labeling with ^18^F. Clinical research showed that [^18^F]-DPA-714 can provide information on the degree of immunosuppression in the TME in a glioblastoma mouse model, and it was concluded that imaging can assist in monitoring chemotherapy treatment outcomes [119, 123]. Wadsworth et al. [124] developed next-generation TSPO radioligands, which resulted in [^18^F]-GE-180 as the most promising candidate. In glioblastoma patients, [^18^F]-GE-180 showed improved tumor-to-background ratios compared to the former generation TSPO-targeting radioligands [125, 126]. Although the uptake was not only caused by TSPO expressing TAMs, as this recent research showed specific uptake in TSPO-expressing tumor cells as well.

Another M2-specific macrophage marker is FRβ [127]. Radioligand imaging of multiple FR isoforms has already been accomplished in 1994 with ^125^I-labeled folate [128]. Over the years, various other folate analogs with different chelators have been developed and optimized [129]. Two have been tested in clinical trials: [^111^In]In-DTPA-folate and [^99m^Tc]Tc-EC20 [130]. These radioligands also bind to the FRα isoform, which can be beneficial since FRα has been found overexpressed on the tumor cell membrane of various cancers [131]. Nevertheless, their lack of FRβ specificity hampers TAM-specific nuclear imaging. A specific anti-FRβ antibody is available [132]; however, no studies have been reported on radiolabeling of this compound.

Furthermore, M2 macrophages exhibit higher endocytic activity compared to M1 macrophages, a process that can be targeted with high-density lipoproteins (HDLs). HDLs have demonstrated increased uptake specific to TAMs [133]. Pérez-Medina et al. [134] synthesized HDL nanoparticles and labeled them with ^89^Zr, resulting in [^89^Zr]Zr-Al-HDL and [^89^Zr]Zr-PL-HDL, which showed high selective uptake for M2 TAMs in a preclinical mouse model. [^89^Zr]Zr-Al-HDL demonstrated the highest uptake of 16.5 ± 2.8%ID/g compared to 8.6 ± 1.3%ID/g for [^89^Zr]Zr-PL-HDL. Unfortunately, the HDL-targeting radioligands also showed high unspecific kidney and bone uptake. Mason et al. [135] developed different ^89^Zr-labeled HDL nanoparticles, and demonstrated that their compounds can be used to quantify the TAM fraction in the TME. In line with this, the HDL nanoparticles could successfully assess the response to a macrophage-targeted therapy.

The scavenger receptor, CD163, is a more M2-specific biomarker than the above-mentioned targets. The presence of CD163-positive TAMs is correlated to a reduced survival in multiple cancers [136]. Unfortunately, not a lot of studies have exploited CD163 for targeted radionuclide theranostics. Presumably, this is because in humans shedding of the receptor is observed, which makes targeting difficult [111, 136]. An anti-CD163 mAb in a rat model demonstrated selective targeting of M2 macrophages; however, no radiolabeled variant has been published yet [137].

### Neo-angiogenic endothelial cells

Tumor angiogenesis is the result of proliferative endothelial cells that form a network of new blood vessels to supply the tumor with oxygen. Hypoxia and acidity in the TME stimulate the release of pro-angiogenic factors, and hereby promote neo-angiogenesis for improved tumor blood flow [138, 139]. Perfusion has been imaged with nuclear medicine for decades by [^15^O]-water PET. However, due to the short half-life of oxygen-15 of 2 min, this technique is only available at centers with a cyclotron [140]. Furthermore, many anti-angiogenic therapies have been developed, and assessing target expression for these therapies by nuclear imaging is very valuable for patient selection [141]. Together, this indicates the need for angiogenesis-targeting radioligands. The most potent targets studied for this include integrins (i.e., αvβ3), aminopeptidase N (APN/CD13), vascular endothelial growth factor receptor (VEGFR), and prostate-specific membrane antigen (PSMA) [142].

**Fig. 6:**
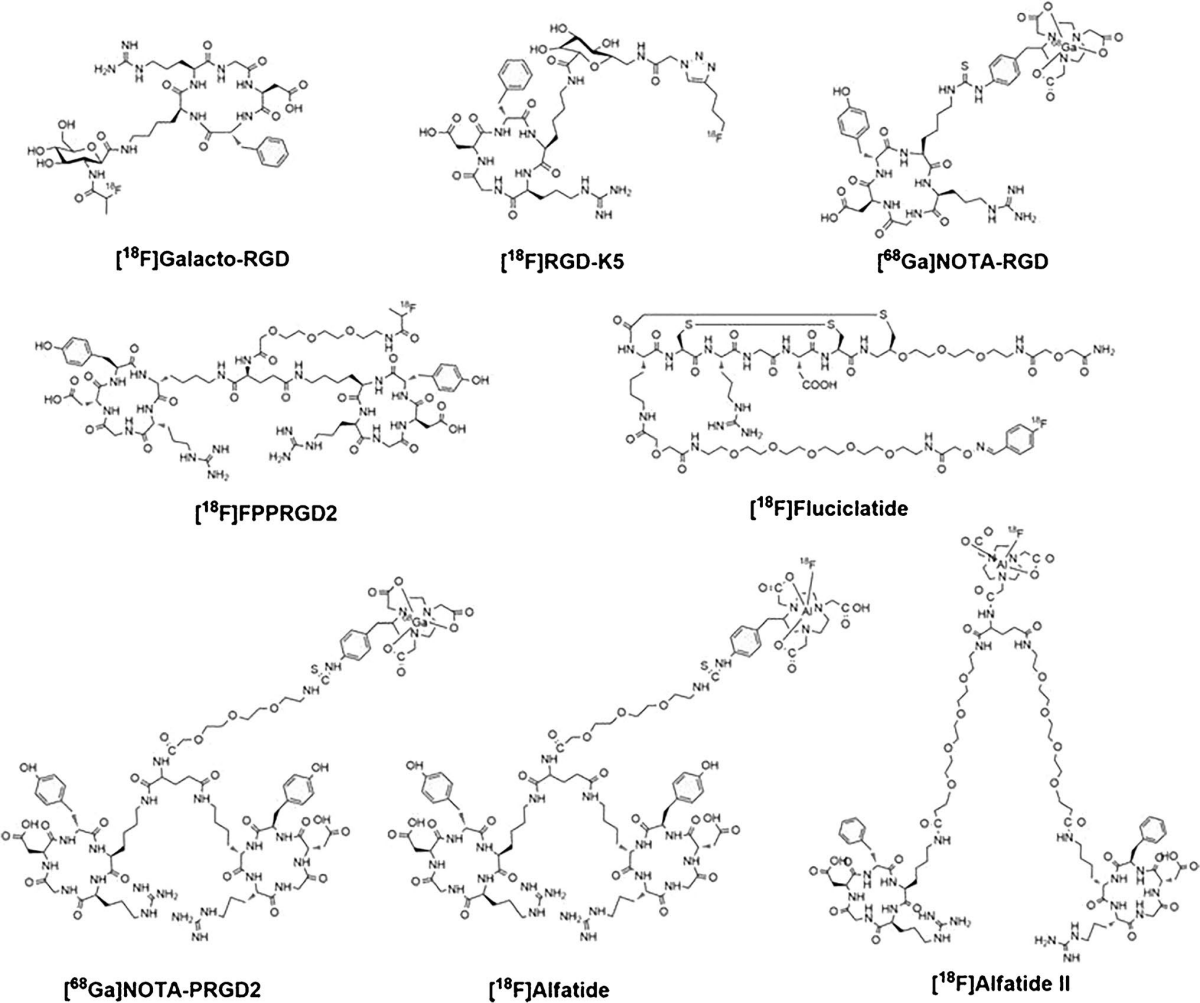
Chemical structures of several monomeric (i.e., [^18^F]Galacto-RGD, [^18^F]RGD-K5, [^68^Ga]NOTA-RGD, [^18^F]Fluciclatide) and dimeric (i.e., [^18^F]FPPRGD2, [^68^Ga]NOTA-PRGD2, [^18^F]Alfatide, [^18^F]Alfatide II) RGD-based PET radioligands. The figure was originally published in Theranostics by Chen et al. [144]. Clinical application of radiolabeled RGD peptides for PET imaging of integrin αvβ3 (2016) Vol. 6(1): 78–92

Integrins are the most researched biomarkers for imaging and therapy of angiogenesis, in particular αvβ3, which is overexpressed by the endothelial cells of almost all tumor vasculature, and on some cancer cells [143]. Most anti-angiogenic radioligands are radiopeptides, based on the Arg-Gly-Asp (RGD) motif, that bind to αvβ3. A wide variety of RGD peptides, labeled with different radioisotopes, have been developed for monitoring response to non-nuclear medicine–based anti-angiogenic therapies [139, 144]. The radioligands are based on the RGDfK or RGDyK structure and are available in cyclic variants, monomers, and multimers. Figure 6 depicts several structures of monomeric and dimeric RGD-based PET radioligands. The most researched and promising RGD-based radioligands include PPRGD_2_, Fluciclatdie, Alfatide I and II, NOTA-RGD and DOTA-RGD, and Galacto-RGD [145]. Of these radioligands, [^18^F]Galacto-RGD was the first αvβ3-targeting PET radioligand applied in the clinic, demonstrating promising tumor-to-background ratios [146]. Research has shown that cyclic RGD peptides have a higher αvβ3 affinity and that multimerization of RGD peptides increases the tumor uptake and retention time. In line with this, cyclic dimers such as FPPRGD_2_ and Galacto-RGD_2_ were developed. [^18^F]FPPRGD_2_ was the first clinically assessed dimer; it was proved to be successful in predicting the early response to anti-angiogenic therapy in a small pilot study with cervical and ovarian cancer patients [147], and in patients with metastatic renal cell cancer [148]. Evidence for improved tumor uptake and retention of cyclic dimers vs. monomers was provided by a clinical study comparing [^18^F]-FPPRGD_2_ and [^18^F]-Galacto-RGD in 8 breast cancer patients. The dimer clearly outperformed the monomer; the SUVmax range was 2.4–9.4 and 0.3–6.8, respectively [149, 150]. Another example of improvement by multimerization is the 7.3-fold increased αvβ3 affinity and 3.9-fold increased preclinical tumor uptake of an RGD trimer, compared to the monomer Galacto-RGD [151]. Unfortunately, increased tumor uptake and retention do not always result in better tumor-to-background ratios, due to a possible simultaneous increase in uptake by healthy tissues. Next to nuclear RGD-targeted imaging of angiogenesis, TRT has also been studied. One example is a theranostic case study in a thyroid cancer patient that underwent [^68^Ga]Ga-DOTA-RGD_2_ pre- and post-treatment with [^177^Lu]Lu-DOTA-RGD_2_, which demonstrated a reduction in metastatic lesions, as well as pain relief [152]_._ In vivo preclinical studies have demonstrated that next to radioligand affinity, αvβ3 activation status determines radioligand uptake [138], stressing the importance of biological understanding of the target. Next to αvβ3, the integrins α5β1, αvβ6, and αvβ8 have been researched for nuclear imaging [153]. However, the developed radioligands for targeting these integrins are less specific.

A different target is APN, which is solely expressed by activated endothelial cells in blood vessels, and is a critical regulator of angiogenesis. Radiopeptides with an Asn-Gly-Arg (NGR) motif are applied for APN targeting. Dénes et al. [154] produced a library of NGR peptides, of which [^68^Ga]Ga-NOTA-c(NGR) showed the best uptake in a cancer mouse model. As seen with RGD peptides, the cyclic and multimeric NGR peptides show an improved binding affinity compared to linear monomers [155]. For instance, the dimeric [^99m^Tc]Tc-NGR_2_ showed improved binding affinity, tumor uptake, and retention over monomeric [^99m^Tc]Tc-NGR_1_ (5.03 ± 0.74%ID/g vs 2.67 ± 0.68%ID/g 4 h p.i. respectively). Unfortunately, this also resulted in a significantly higher liver uptake of the dimer (9.07 ± 0.67%ID/g vs 7.85 ± 0.96%ID/g 4 h p.i. respectively) [156]. To compare αvβ3 and APN targeting modalities, ^99m^Tc-labeled cyclic NGRyK and RGDyK have been evaluated in a melanoma mouse model. The comparison demonstrated significantly higher tumor uptake of the RGDyK compared to NGRyK (7.85 ± 2.34%ID/g vs 1.07 ± 0.23%ID/g) [157]. In an attempt to optimize tumor targeting, a [^68^Ga]Ga-NGR-RGD heterodimer with an affinity for both APN and αvβ3 was developed. As expected [^68^Ga]Ga-NGR-RGD demonstrated better targeting efficiency than the NGR and RGD monomers [158]. Even though preclinical results with NGR-targeting radiopeptides are promising, studies have demonstrated that these peptides are very unstable due to their susceptibility for non-enzymatic degradation. For this reason, no clinical trials have been performed with NGR radiopeptides to date [154].

**Fig. 7:**
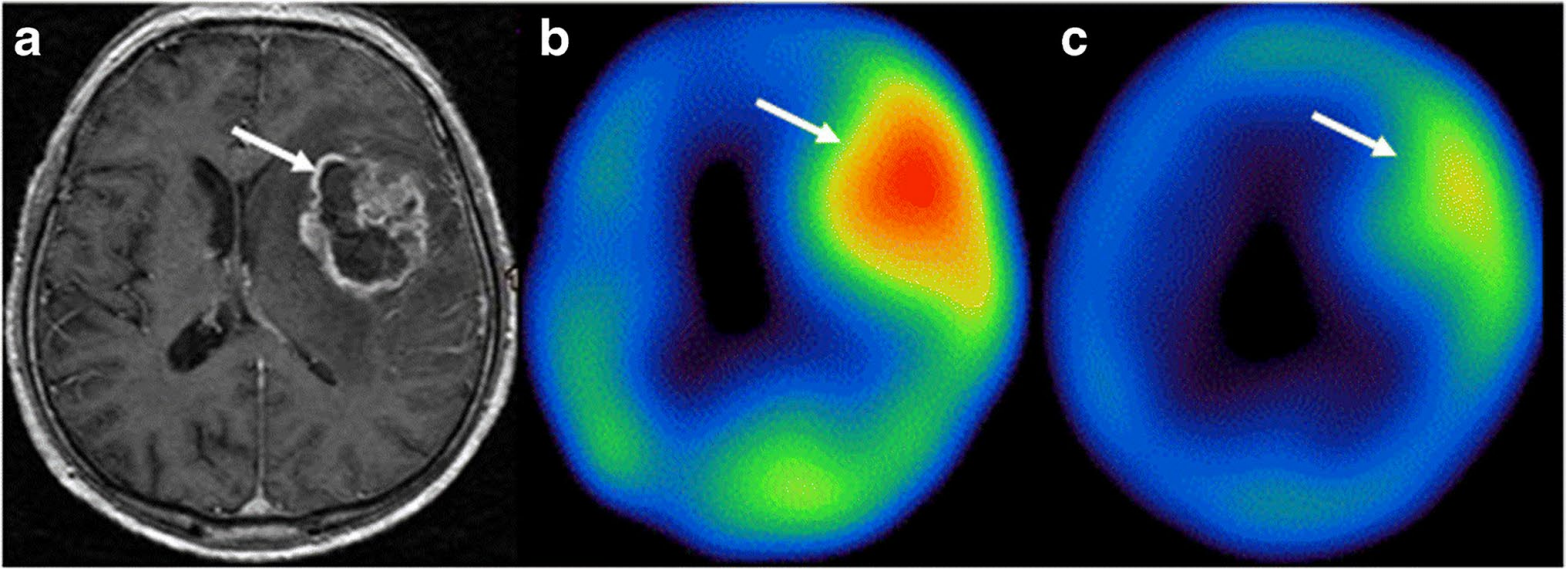
[^123^I]I-VEGF_165_ SPECT/MRI of a grade IV glioma in a 60-year-old patient. **a** Acial T1-weighted MRI for anatomical reference of the tumor lesion. **b** [^123^I]I-VEGF SPECT imaging before radiation therapy and **c** lower radioligand accumulation 1 week post-radiation therapy. White arrows indicate the tumor lesion. The figure was originally published in EJNMMI by Rainer et al. [163]. The prognostic value of [^123^I]-vascular endothelial growth factor ([.^123^I]-VEGF) in glioma (2018) Vol. 45(13): 2396–2403

Another potent target for angiogenesis, expressed by vascular tumor endothelial cells, is the VEGFR. More than 10 VEGF(R)-targeted drugs are FDA-approved [159] and there is a need for a non-invasive method to select patients eligible for these treatments. Radiolabeled anti-VEGF antibodies, VEGF radiopeptides, and small-molecule inhibitors have been evaluated for imaging, and some for therapy [160]. A potent example of an anti-VEGF mAb radioligand is radiolabeled bevacizumab. Bevacizumab has been labeled with ^111^In and ^99m^Tc for imaging and with ^177^Lu for therapy. Labeled with all aforementioned isotopes, bevacizumab demonstrated selective and stable tumor uptake in preclinical studies. Unfortunately, the radioligand is also associated with high uptake in healthy tissues, which needs to be resolved before clinical studies can be considered [161, 162]. Next to mAbs, VEGF radiopeptides have been explored, with the VEGF_121_ and VEGF_165_ isoforms being the most promising. [^123^I]I-VEGF_165_ scintigraphy in 23 patients with brain tumors allowed for visualization of gliomas and correlation with disease grade [163]. However, PET/SPECT images obtained with radiolabeled VEGF ligands can be distorted by endogenous VEGF expression of cancer cells, resulting in VEGFR saturation [164]. Lastly, VEGFR targeting was accomplished with radiolabeled small-molecular inhibitors of VEGFR tyrosine kinase, with high target affinity. Particularly radiolabeled versions of the clinically approved sunitinib and sorafenib have been extensively researched for both SPECT and PET imaging in preclinical studies. So far, no clinical trials have been reported [160]. Figure 7 shows an example of [^123^I]I-VEGF_165_ imaging before and after external beam radiation therapy in a patient with grade IV glioma.

More recently, it has been discovered that PSMA is upregulated on the endothelial cells of the neo-vasculature in over 70% of solid tumors [165]. PSMA radioligands PSMA-11, PSMA-617, and PSMA-I&T have been extensively researched in prostate cancer, and this experience can be beneficial to translate its use to target neo-angiogenesis in other solid cancers. In a triple-negative breast cancer cell line and xenograft mouse model, the application of [^177^Lu]Lu-PSMA-617 impaired blood vessel formation, which is an encouraging result [166].

Next to the above, vascular endothelium-cadherin [167], PDGFRβ, endoglin [168], or phosphatidylserine [169] are less researched molecular targets of neo-angiogenesis for radionuclide imaging and therapy [170].

### Tumor-infiltrating lymphocytes

TILs are supposed to exhibit an inflammatory response to clear cancer. However, through cytokine secretions and ligand interactions, the tumor can evade the immune system, creating an immunosuppressive environment that aids cancer progression [171]. In line with this, immune checkpoint inhibitors are currently one of the most researched novel anti-cancer treatments. Nuclear imaging can be crucial for selecting patients that will benefit from these immunotherapies. After immunotherapy, [^18^F]FDG PET/CT scans show pseudo-progression caused by the enhanced immune infiltration, which cannot be distinguished from tumor growth. Therefore, TIL-targeting nuclear imaging can help visualize the treatment response more accurately, and in addition monitor the immune status of cancers [172]. It has been proven challenging to find a molecular marker that is suitable to specifically target TILs, because of their conflicting roles (i.e., pro- and anti-tumorigenic), and because marker expression adapts when TILs mature [172]. Radioligands have been developed targeting programmed cell death (PD1) and PD ligand (PDL1) interactions, and the chemokine receptor 4 (CXCR4) and its ligand C-X-C motif chemokine ligand 12 (CXCL12).

The PDL1/PD1 axis is a promising target because immune checkpoint inhibitors against PDL1/PD1 have shown great results as an anti-cancer treatment, and there is an urgent need to develop radioligands targeting PDL1/PD1 to select potential responders. Radiolabeled antibodies against PDL1 have been clinically evaluated, including [^89^Zr]Zr-atezolizumab and [^89^Zr]Zr-avelumab [173, 174]. [^89^Zr]Zr-atezolizumab uptake could be related to atezolizumab treatment response in lung cancer patients [174]. In addition, anti-PD-1 antibodies, including ^89^Zr-labeled pembrolizumab and nivolumab, have been evaluated. Clinical trials in melanoma and non-small cell lung cancer patients showed that the uptake of both these radioligands correlates with patient responses to anti-PD-1 checkpoint inhibition therapy with their corresponding inhibitor [175, 176]. Notably, studies showed that the level of PDL1 expression determined by immunohistochemistry does not accurately predict the response to therapy. This illustrates the need for radiolabeled PDL1 and PD1 antibodies for improved response prediction. Even though all the antibodies mentioned above show good results in clinical pilot studies, smaller ligands can have advantageous pharmacokinetics. In line with this, smaller ligands have been evaluated in clinical trials, such as anti-PDL1 sdAb [^99m^Tc]Tc-NM-01 (NCT02978196) and [^18^F]F-BMS-986192 [177]. Of these, [^99m^Tc]Tc-NM-01 demonstrated faster clearance than the radiolabeled antibodies in lung cancer patients [176].

The CXCR4 and its ligand CXCL12 affect the tumor immune status. CXCR4 is commonly overexpressed on macrophages, lymphocytes, neutrophils, and other hematopoietic cells in the TME. These CXCR4-expressing cells are attracted to areas with high CXCL12 expression, hereby directing immune cell trafficking in the TME. Moreover, cancer cells hijack this system by overexpressing CXCR4, allowing for rapid metastasizing to distant organs with high CXCL12 expression [178, 179]. Altogether, CXCR4 is an appealing target for therapy and various CXCR4-targeting imaging and therapeutic radioligands have been designed. Three classes of compounds are relevant for nuclear interventions directed at CXCR4; that is FC-131-based penta-peptides, radiolabeled cyclam derivatives, and T140 analogs [180].

**Fig. 8:**
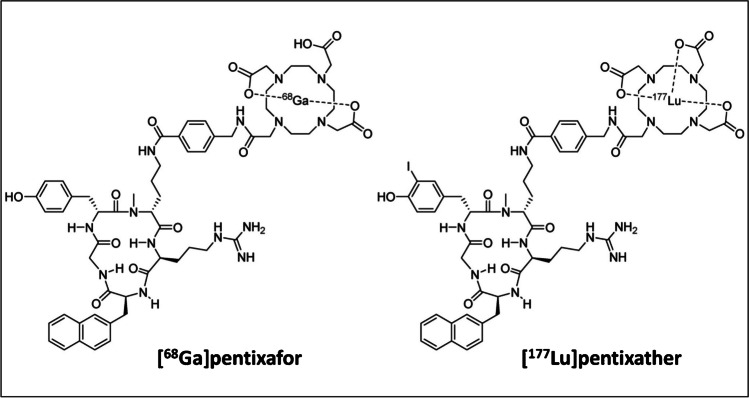
Chemical structures of [^68^Ga]Ga-Pentixafor and [^177^Lu]Lu-Pentixather. Originally published in Theranostics by Schottelius et al. [185]. [.^177^Lu]pentixather: Comprehensive Preclinical Characterization of a First CXCR4-directed Endoradiotherapeutic Agent (2017) Vol. 7(9): 2350–2362

The most successful FC-131-based radioligand is [^68^Ga]Ga-Pentixafor, which showed superior imaging results compared to [^18^F]FDG in a clinical pilot study with multiple myeloma patients [181]. In a pilot study with esophageal cancer patients, [^68^Ga]Ga-Pentixafor imaging was feasible; however, heterogeneous CXCR4 expression limited its application in this cancer type [182]. As Pentixafor does not allow for labeling with therapeutic isotopes, the structurally closely related Pentixather was developed. Since CXCR4 is overexpressed on the surface of hematological cancer cells, ^177^Lu- and ^90^Y-labeled Pentixather have been evaluated as adjunct to high-dose chemotherapy in this cancer type. This resulted in a more beneficial outcome of the combination therapy vs monotherapy with the radioligand [183, 184]. In addition, a case study in B-cell lymphoma patients demonstrated the possibility of a theranostic approach with [^68^Ga]Ga-Pentixafor and [^177^Lu]Lu-Pentixather [185]. No therapeutic or theranostic studies have been performed with CXCR4 radioligands targeting the TME. However, the above studies indicate the potential of [^68^Ga]Ga-Pentixafor and [^177^Lu]Lu-Pentixather theranostics for targeting CXCR4-positive stromal cells. Furthermore, it should be noted that even small structural differences can affect compound behavior, and in this particular case, a difference in radioligand accumulation of [^68^Ga]Ga-Pentixafor and [^177^Lu]Lu-Pentixather in healthy organs (i.e., liver, spleen, kidneys) was observed. The (differences in) chemical structures of [^68^Ga]Ga-Pentixafor and [^177^Lu]Lu-Pentixather are shown in Fig. 8.

Next, T140 peptide analogs showing high CXCR4 affinity have been considered for CXCR4-targeting nuclear medicine–based interventions. The most promising compound is NOTA-NFB; a modified T140 radiopeptide, specifically developed to improve pharmacokinetics and enable radiolabeling. In a pilot trial with glioblastoma patients, [^68^Ga]Ga-NOTA-NFB showed specific tumor accumulation, and favorable SUVmax compared to [^18^F]FDG PET. Although, the reported effective radiation dose to healthy tissues of [^68^Ga]Ga-NOTA-NFB exceeded that of formerly mentioned [^68^Ga]Ga-Pentixafor (25.4 µSv/MBq vs. 15.3 µSv/MBq respectively) [186, 187].

Lastly, the FDA has approved non-radiolabeled bicyclam AMD3100 (Plerixafor/Mozobil) for anti-CXCR4 therapy. For the patient selection of this therapy, radiolabeling of AMD3100 has been performed with a variety of radioisotopes, including ^68^Ga, ^64^Cu, and ^111^In [188, 189]. In addition, the improved monocyclam AMD3465 was developed and radiolabeled. Preclinical in vivo studies demonstrated improved pharmacokinetics of [^64^Cu]Cu-ADM3465 compared to [^64^Cu]Cu-AMD3100. Unfortunately, both compounds, as well as their derivatives, showed high liver uptake [190, 191]. To exclude if the copper isotopes are responsible for the observed liver uptake, Brickute et al. [190] developed an ^18^F-labeled AMD3465-derivative (MCFB). [^18^F]F-MCFB showed selective tumor detection; however, high liver uptake was still observed. This hampers the translation of these radiolabeled cyclam derivatives to the clinic.

Apart from the above, radioligands targeting specific immune cell types have been developed; however, we consider this beyond the scope of this review.

### Cancer-associated adipocytes

CAAs are crucial for storing and providing energy to cancer cells, which is necessary for tumor growth and metastasizing [192]. Cancer cells show a higher dependency on fatty acids produced by adipocytes than healthy cells. Radioligand targeting of CAAs is still a very unexplored field. However, CAAs provide a multi-cancer target for nuclear imaging or could even serve as a target for TRT. A few studies evaluated this concept by evaluating radioligands targeting fatty acid–binding protein 4 (FABP4) or the increased expression of the key enzyme fatty acid synthase (FASN) [193].

FABP4 plays a critical role in the interaction between cancer cells and CAAs. It was found to be elevated at the boundary between tumor cells and CAAs. Temma et al. [194] developed FABP4-targeting TAP1 and radiolabeled it with ^125^I and ^18^F for SPECT and PET imaging, respectively. [^18^F]F-TAP1 showed a tumor uptake of 3.86 ± 0.39%ID/g, and an uptake in the blood of 10.38 ± 0.56%ID/g (3 h p.i.), resulting in a very poor tumor-to-blood ratio (0.37 ± 0.05). The observed high blood signal is a limiting factor for imaging analyses. The iodine-125 labeled version of TAP1 showed lower, but more rapid tumor uptake, and lower blood levels than [^18^F]F-TAP1. Despite the lower blood levels, [^125^I]I-TAP1 tumor-to-blood ratios remained poor (0.42 ± 0.04), and in addition, a very high kidney uptake was observed [195]. Another group developed the FABP4 inhibitor [^14^C]C-BMS-309403 which showed reasonable target affinity; however, no in vitro or in vivo results are reported [196]. Next to CAAs, FABP4 expression is found on TAMs and a variety of cancer cells; thus, radioligand uptake is not CAA specific [197].

Cancer cells are metabolic-dependent on increased fatty acid production facilitated by CAAs. FASN facilitates in this, and is overexpressed in CAAs in the TME [198]. ^11^C-labeled acetate has been researched to determine FASN expression levels in vivo in prostate cancer xenografted mice. Radioligand uptake could be related to FASN expression; however, the uptake was very low (0.276 ± 0.05%ID/g) and high radioligand uptake in normal tissues was observed [198].

### Summary: targeting the cellular components of the TME

As described above, there is a wide variety of molecular markers available on the cellular TME components for nuclear imaging and TRT (Table 2). Radioligands targeting the cellular components of the TME can play an important role in patient selection and treatment response monitoring. The above-described radioligands that are far in clinical development are often aided by a well-defined target. This is demonstrated by radiolabeled Pentixafor/Pentixather and PD(L)1 inhibitors for imaging of TILs, and RGD-targeting radioligands for imaging of neo-angiogenesis. Moreover, the recent discovery that RGD-targeting radioligand uptake is also affected by the αvβ3 activation status, and not solely by its expression [138], further illustrates the importance of biological understanding for the development of improved radioligands.

**Table 2:** An overview of the targets available on cellular TME components, with a number of their corresponding radioligands

Source	Target	Compound	Research phase	Reference
**Cancer-associated fibroblasts**	FAP	FAPI-04 FAPI-46 FAPI-74 FAPI-2286 OncoFAP BiOncoFap DOTA-2P(FAPI)_2_ DOTAGA.(SA.FAPi)_2_	Clinical trial Clinical trial Clinical trial Clinical trial Clinical trial Preclinical Preclinical Preclinical	[92, 95, 100, 104, 106–108]
**Tumor-associated macrophages**	MMR (CD206)	γ-Tilmanocept NOTA-anti-MMR-sdAb Man-LIPs	Approved Clinical trial Preclinical	[113, 115, 133]
	TSPO	RPK11195 PBR28 DPA-714 GE-180 DPA-713	Clinical trial Clinical trial Clinical trial Clinical trial Preclinical	[122, 123, 125]
	Folate receptor β	DTPA-folate EC20	Clinical trial Clinical trial	[129, 130]
	Elevated endocytosis	Al-HDL PL-HDL	Preclinical Preclinical	[134, 135]
	CD163	Anti-CD163 mAb^b^	n/a	[137]
**Neo-angiogenic endothelial cells**	Perfusion	^15^O-water	Approved	[140]
	αvβ3 Integrin	Galacto-RDG FPPRGD_2_ Fluciclatide Alfatide Alfatide II NOTA-RGD_2_ DOTA-RGD_2_	Clinical trial Clinical trial Clinical trial Clinical trial Clinical trial Clinical trial Clinical trial	[145, 152]
	APN/CD13	DOTA-NGR_1/2_ cNGR NGRyk	Preclinical Preclinical Preclinical	[154, 155]
	VEGF/VEGF-R	VEGF_165_ VEGF_121_ Bevacizumab	Clinical trial Preclinical Preclinical	[160, 163]
	PSMA	PSMA-11 PSMA-I&T PSMA-617	Approved^c^ Clinical trial Clinical trial	[165, 166]
**Tumor-infiltrating immune cells**	PDL1 (tumor/stromal cells)	Atezolizumab Avelumab NM-01 BMS-986192	Clinical trial Clinical trial Clinical trial Clinical trial	[174, 176, 177]
	PD1 (immune cells)	Nivolumab Pembrolizumab	Clinical trial Clinical trial	[175]
	CXCR4/CXCL12	Pentixather/Pentixafor NOTA-NFB AMD3100 AMD3465	Clinical trial Clinical trial Preclinical Preclinical	[185–187, 190]
**Cancer-associated adipocytes**	FABP4	TAP1 BMS-309403	Preclinical Preclinical	[194–196]
	FASN	Acetate	Preclinical	[198]

^b^Affinity for rat CD163, tested in arthritis, not cancer. ^c^FDA-approved for treatment of prostate cancer, not in angiogenesis setting

However, in the case of anti-TAM radioligands, extensive biological information is available, but a radioligand specific for an M2 marker has yet to be developed. The FRβ and CD163 were identified as highly specific M2 TAM markers; however, no radioligands specifically targeting these biomarkers have been reported yet. Thus, next to the understanding of the target biology, the development of highly specific radioligands is crucial for the success of nuclear imaging and TRT.

In contrast to TAMs, there is still less known about CAFs and CAAs. This is also reflected in the development of radioligands for targeting these TME components. Regarding CAFs, recently, there is a relatively high nuclear research output and the FAP-targeting radioligands are extremely promising for pan-cancer nuclear imaging. However, it has proven difficult to increase tumor retention for effective TRT, and more knowledge on FAP behavior is needed to overcome this problem. In addition, only few studies described the use of radioligands for targeting the TME (i.e., a VEGF- and FAP-targeting radioligand) of intracranial tumors, mostly likely because there is a lack of understanding on the mechanisms behind radioligands passing the blood–brain barrier. More and better understanding hereof will not only be of benefit for TME-targeting radioligands, but for radioligands in general.

Whereas the processes described in the first section are mainly interesting for imaging, the above-described molecular markers of the cellular TME components have shown to be more promising for both nuclear imaging and TRT in a multi-cancer setting.

## Future perspectives for TME-targeting radionuclide interventions

A radioligand directed towards a universally expressed tumor biomarker allows for broad application of nuclear imaging and therapy. In contrast to biomarkers expressed by cancer cells, TME biomarkers are more universally available across different cancer types. Thus, radioligands directed against these biomarkers have multi-cancer, or even pan-cancer, applications. A single-cell RNA and protein analysis showed that 46/68 stromal cell populations overlap between different cancer types [2], indicating that there are many shared TME phenotypes between solid cancers that provide biomarkers for tumor targeting. In line with this, relevant TME biomarkers have been identified and radioligands targeting these biomarkers have been developed and evaluated, some only preclinically while others have also been applied clinically. From these studies, it has become clear that there are a few challenges that need to be overcome to accelerate the development and to promote clinical translation of (novel) TME-targeting radioligands.

First, since only a few of the explored TME targets are suitable for pan-cancer targeting (i.e., hypoxia, CAFs, and angiogenesis), and the majority has multi-cancer applications, it is relevant to identify which cancer types can benefit the most from the application of a specific TME-targeting radioligand. Next, it is known that external beam radiation can induce radioresistance and senescence, and promote angiogenesis and matrix stiffness in the TME [199]. Little is known about the effect of local radiation with TRT on the TME, and this should be explored for the correct application of radioligands for treatment purposes. In addition, knowledge of the bystander effect, which is the effect irradiated cells indirectly exhibit on surrounding cells, is also important to understand how radiation delivered to the TME will affect the cancer cells. Lastly, one of the most pressing issues in the TME is that specific cell types often exist in pro-tumorigenic or anti-tumorigenic forms, e.g., CAFs and TAMs [87]. Regarding TAMs, the ability to differentiate between anti-tumorigenic M1 and pro-tumorigenic M2 TAMs underscores the importance of identifying subtypes, which can promote the development of very specific pro-tumorigenic-targeted agents [109]. Altogether, an increased (radio)biological understanding of the TME is necessary to aid research on novel TME-targeting radioligands and promote their clinical translation.

Partly due to the lack in (radio)biological understanding, most of the investigated TME-targeting radioligands are researched for imaging, and not (yet) for therapy. As solely nuclear imaging agents, these radioligands can play a role in patient selection for non-nuclear medicine–based anti-cancer interventions; thus, they are used in interdisciplinary theranostic applications. Examples are imaging by RGD-based radioligands for the selection of anti-angiogenic therapies, and radiolabeled checkpoint inhibitors for identifying patients suitable for treatment with the corresponding PD1/PDL1 immune checkpoint inhibition therapy [200]. This multidisciplinary theranostic strategy allows for quick redirection and adjustment of treatment schedules when necessary, which is especially relevant for TME markers of which expression is highly variable between patients. In contrast to radioligands targeting TME biomarkers that are only expressed in subset of patients, e.g., CAIX or PD1/PDL1, the developed radioligands targeting hypoxia, the ECM, CAFs, and CAAs are suitable for more universal nuclear imaging of solid tumors. These radioligands could potentially offer a valuable addition to the current gold standard [^18^F]FDG PET scans. However, clinical experience for improved scan interpretation is needed to determine whether a decrease in radioligand uptake should be interpreted as a treatment response and consequent tumor shrinkage, or only as depletion of the corresponding TME component.

In addition to radioligands that predict the response to non-radioactive anti-cancer therapies, radioligands that can be used for both nuclear imaging and TRT, and thus for nuclear medicine–based theranostics, have also been developed. Currently, the most potent targets in the TME for nuclear imaging and TRT are TNC and FAP. In addition, αvβ3 integrin, CXCR4, TSPO, and FRα/β have been found to be expressed by both stromal cells and cancer cells making them less TME-specific, but nevertheless an appealing target for nuclear medicine–based theranostics [125, 131, 143, 178]. Furthermore, TRT directed at the TME was studied as a combination treatment in one case, in which^177^Lu/^90^Y-labeled Pentixather was administered adjunct to high-dose chemotherapy [184]. In the past, TRT has proven to be promising as a combined modality for cancer treatment [201], and thus treatment strategies combining TME-directed TRT, with other anti-cancer therapies are worth exploring. Especially, since the TME can act as a protective barrier against anti-cancer treatment, TME-directed radionuclide therapy could offer a solution, by paving the way for other anti-cancer treatments to reach the tumor cells. However, for this, more TME-targeting radioligands for TRT first need to be developed and evaluated.

In conclusion, TME-targeting radioligands are promising for nuclear imaging and TRT with multi-cancer, and even pan-cancer application. Next to imaging, there should be more focus on the possibilities for TRT and nuclear medicine–based theranostics. The growing (radio)biological understanding and the increasing research output on nuclear medicine–based interventions will hopefully stimulate the development and application of TME-targeting radioligands. We are confident that TME-targeted radionuclide interventions will offer a valuable strategy for improved cancer patient care.
